# Supplementary material for: All-at-once RNA folding with 3D motif prediction framed by evolutionary information
Source: Nat Methods. 2025 Oct 3;22(10):2094–106. doi: 10.1038/s41592-025-02833-w (PMC12510878; doi:10.1038/s41592-025-02833-w)
Supplement: Supplementary file 3 — Source code to run CaCoFold-R3D. Data and results. [file 41592_2025_2833_MOESM3_ESM.gz › supplemental_material/results/CaCoFold-R3D/CaCoFold-R3D_Rfam/RF00262_sar.R2R.sto.pdf]

RF00262_sar

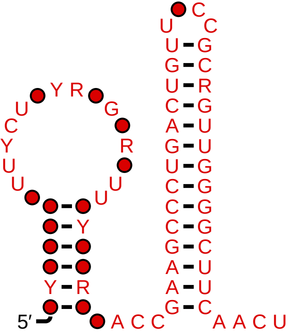

C
U C
U G
G C
U R
C G
A U
G U
U G
C G
C G
C G
G C
A U
A U
G C
Y R
U G
C R
Y
U U
U U
Y
Y R
5′
A C C
A A C U
